# Is military employment associated with the risk of cognitive impairment in older adult? Evidence from China

**DOI:** 10.3389/fepid.2026.1873441

**Published:** 2026-07-23

**Authors:** Ren-Ping Gu, Wen-Bao Liu, Xiang-Dong Peng, Ai-Yong Zhu

**Affiliations:** 1 School of Nursing and Health Management, Shanghai University of Medicine and Health Sciences, Shanghai, China; 2 Faculty of Military Health Service, Naval Medical University, Shanghai, China

**Keywords:** aged, cognition, congitive functions, military personnel, veterans

## Abstract

**Background:**

It remains unknown whether a link exists between military employment and alterations in cognitive function later in life.

**Aim:**

To examine the association between military employment and cognitive impairment in adults.

**Methods:**

Chinese Longitudinal Healthy Longevity Survey data from 2018 were retrospectively analyzed. The participants included Chinese military veterans and non-veterans aged ≥65 years. Veteran status was defined according to the self-reported prior service as uniformed military personnel in the armed forces. Cognitive function was assessed using the Chinese version of the Mini-Mental State Examination. Nearest neighbor caliper matching without replacement was conducted to generate matched pairs. Binary logistic regression models were used to examine the association between military employment and cognitive impairment in the matched cohort, with adjustments for age, sex, education level, marital status, current smoking status, and chronic disease history. To assess the robustness of the findings, sensitivity analyses were conducted by defining cognitive impairment according to higher education-specific cut-off values.

**Results:**

Data from 102 matched pairs were analyzed. The logistic regression analyses demonstrated that military employment was not significantly associated with the risk of cognitive impairment (odds ratio (OR) [95% confidence interval (CI)]: 0.61 [0.24–1.55). The sensitivity analyses yielded similar results.

**Conclusions:**

There was no evidence to suggest military employment affected the risk of cognitive impairment later in life in this Chinese cohort. Nevertheless, owing to the high prevalence of impaired cognition among Chinese veterans, it remains an important health condition requiring further study.

## Introduction

The progressive increase in life expectancy and population aging have led to greater research interest into cognitive impairment, including dementia, among the elderly, and overcoming the condition represents a difficult public health challenge (1). Cognitive impairment refers to dysfunctional performance in one or more cognitive domains, such as memory, language, executive function, attention, and motor function (2). According to the Global Burden of Diseases, Injuries, and Risk Factors Study (GBD), the number of individuals with dementia is expected to increase from 57.4 million globally in 2019 to 152.8 million in 2050. In China, specifically, the number of people with dementia is expected to increase twofold by 2050 (3). For people aged 60 years, the prevalence of dementia in China was estimated to be 6.0%, representing 15.07 million adults aged 60 years or older with dementia (4).

Previous studies have shown that cognitive decline and dementia in older people are associated with multiple factors, including, but not limited to, advanced age, female sex, low levels of education, and history of hypertension, hyperlipidemia, diabetes, and heart and cerebrovascular diseases (4, 5).

Older adults with prior military service may face an elevated risk of cognitive impairment in later life. Several risk factors associated with cognitive impairment that have been identified in the general population may be more prevalent among military veterans. For example, vascular risk factors, which increase the overall risk of dementia, appear more common in veteran populations compared to the general public (6). Furthermore, data from the 2010 Behavioral Risk Factor Surveillance Survey indicated that male veterans were more likely than male civilians to report current smoking and heavy alcohol consumption (7). Military experience may also be associated with a higher likelihood of exposure to events that can significantly increase cognitive health risks. For example, traumatic brain injury (TBI) and posttraumatic stress disorder (PTSD) are particularly common among military personnel owing to their unique set of exposures (8). One study showed that in a sample of active-duty U.S. military service members, cognitive impairment associated with TBI adversely affected performance in tasks related to working memory and basic neural processing (9). A large national study of U.S. military veterans with TBI demonstrated that mild TBI, even without loss of consciousness, was associated with a more than twofold increase in the risk of dementia (10). Recent research has also shown that veterans with comorbid TBI and cannabis use disorder (CUD) may be more susceptible to early-onset cognitive disorders, including dementia (11). The prevalence of PTSD is also high among service members and veterans, and those with PTSD are more likely than those without the disorder to meet the criteria for memory impairment and to exhibit impairment in any cognitive domain (12). A recent meta-analysis quantifying the association between PTSD and the risk of dementia demonstrated that PTSD is a strong risk factor for all-cause dementia in both military and non-military populations (13). Overall, these military-associated factors may contribute collectively to the development of cognitive impairment.

Although the relationship between military service and cognitive outcomes is not unidirectional, a competing body of evidence suggests that military service may confer cognitive advantages. It is possible that the healthy soldier effect (HSE) can lead to better overall health later in life. The HSE, which is the military equivalent of the ‘healthy worker effect’, is a term commonly used to describe the phenomenon in which lower mortality rates are observed in veterans compared to those in the general population (14, 15). Typically, recruitment into the Armed Forces requires pre-service assessments to identify individuals with good physical health (16). Furthermore, the emphasis on physical fitness during service and better healthcare received during and after service are attributable to the HSE (17). Thus, it is possible that the prevalence of cognitive impairment among elderly veterans may be lower than that of the general population.

It remains unclear, however, whether military employment can provide benefits that protect against the development of cognitive dysfunction, particularly in the Chinese veterans. Therefore, the aim of this study was to examine the association between military employment and cognitive impairment in a sample of elderly Chinese individuals with and without prior military service. More specifically, the study investigated whether veterans were more likely than non-veterans to experience cognitive impairment.

## Methods

### Study population

Data from the Chinese Longitudinal Healthy Longevity Survey (CLHLS) were retrospectively analyzed (18). Half of the counties and cities in 22 of China's 31 provinces, covering approximately 85 percent of the total population of China, were randomly selected. The CLHLS targeted elderly individuals aged 65 years and older, as well as younger adults aged 35–64 years, covering different occupational groups. Important variables that were assessed included health status, disability, death and survival outcomes, demographic characteristic, family-related factors, socioeconomic factors, income level, needs and costs related to care of the elderly, and behavioral risk factors associated with mortality and healthy aging. One unique feature of the CLHLS is that relatively comprehensive information on the extent of disability and suffering before dying was obtained from close family members in cases in which the interviewees had died before the subsequent wave (19, 20). Trained interviewers conducted face-to-face surveys in participants’ residences using standardized questionnaires. The surveys are administered in participants’ homes by trained interviewers with a structured questionnaire. Questions regarding cognitive function are obtained directly from participants during these structured interviews. The CLHLS study was approved by the Research Ethics Committee of Peking University (IRB00001052-13074). Written informed consent was obtained from all study participants and their proxy respondents.

**Figure 1:**
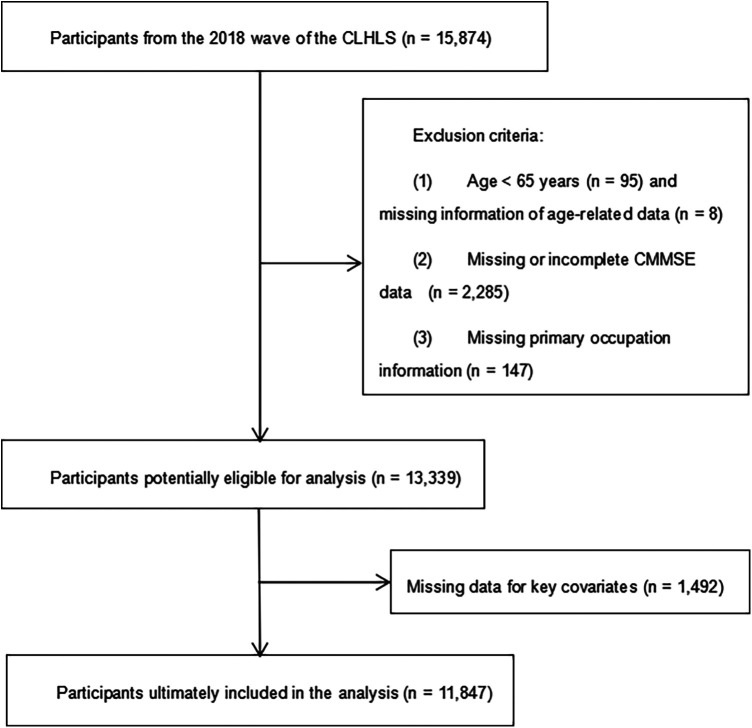
Flow chart of the selection and analysis of the study participants (is military employment associated with the risk of cognitive impairment? Evidence from China, Shanghai, China, 2025).

The present study analyzed data collected from the 2018 wave of the CLHLS (*n* = 15,874). The exclusion criteria were as follows: (1) age < 65 years (*n* = 95) and missing information of age-related data (*n* = 8); (2) missing or incomplete data related to the Chinese version of the Mini-Mental State Examination (CMMSE) (*n* = 2,285); and (3) missing information pertaining to the primary occupation (*n* = 147). Additionally, 1,492 participants with missing data for key covariates were excluded. Ultimately, 11,847 participants were included in the study. Figure 1 shows a flow chart outlining the selection and analysis of the study participants.

### Assessment of cognitive impairment

Cognitive function was evaluated using the standardized CMMSE instrument, which systematically measures: 1) orientation (time/place), 2) immediate verbal recall, 3) attention and serial calculation, 4) delayed memory retrieval, and 5) language production with visual construction abilities (19). The total CMMSE score ranges from 0 to 30 points, with higher scores being indicative of better cognitive function. Based on prior literature, cognitive impairment was identified based on education-specific CMMSE cut-off scores (≤16 points for those without schooling; ≤19 points for those with 1–6 years of education; and ≤23 points for those with > 6 years of education). The established cut-off values demonstrated robust diagnostic accuracy, with sensitivity ranging from 87.6% to 94.3% and specificity spanning 80.8% to 94.3% (21).

### Military employment

Based on the data from the 2018 wave of the CLHLS, military employment status was determined by asking the participants what their primary occupation was before retirement. Participants were classified as having military employment if they reported that their main occupation before retirement was military personnel.

### Covariates

Ten covariates were selected as potential confounders based on previous literature (4, 5). The sociodemographic factors included age (continuous), sex (male or female), level of education (years of schooling: < 1 year; ≥1 and ≤6 years; and > 6 years), and marital status (married, separated, divorced, widowed, or never married). Lifestyle factors included the current smoking status (yes or no). Finally, self-reported assessments of the history of chronic diseases included hypertension (yes or no), hyperlipidemia (yes or no), diabetes (yes or no), heart disease (yes or no), and cerebrovascular disease (yes or no).

### Statistical analysis

Propensity score matching was conducted to generate 102 matched pairs using nearest neighbor caliper matching (0.20 times the standard deviation (SD) of the logit of the propensity score) without replacement (22, 23). Ten covariates included in the propensity score estimation model were age, sex, level of education, marital status, current smoking status, hypertension, hyperlipidemia, diabetes, heart disease, and cerebrovascular disease. Descriptive statistics were used to describe participants’ characteristics. Categorical data are presented as percentages and frequencies, whereas continuous data are expressed as means and SDs. Prior to matching, between-group differences in baseline characteristics were compared using independent-samples t-tests and chi-square tests; after matching, paired t-tests and McNemar tests were employed for balance assessment, in recognition of the paired data structure. Binary logistic regression models were used to examine the association between military employment and cognitive impairment in the matched cohort. However, the potential interaction between age and hypertension was not examined. In addition, sensitivity analyses were conducted based on the higher education-specific cutoff values for the CMMSE scores. As a supplementary analysis, cognitive function was examined as a continuous outcome measured by the CMMSE score using multiple regression analysis to examine the relationship between military employment and cognitive impairment (Supplementary 1). Statistical analyses were performed using R software (version 4.5.0), with statistical significance based on *α* = 0.05 (two-sided).

## Results

Two matched groups of elderly participants were generated, including a group of 102 veterans and a group of 102 non-veterans (Table 1). Consequently, 11,640 non-veterans were discarded and excluded from the subsequent outcome analysis. The distributions of propensity scores in the veteran and non-veteran groups before and after matching were inspected graphically using kernel density plots (Supplementary 2). After matching, the propensity score distributions were well balanced between the groups. Covariate balance between the matched groups was evaluated by calculating standardized mean differences (SMDs) for all variables included in the propensity score model. Following matching, all but three post-matching SMD values were below the prespecified balance threshold of <0.10. In the matched cohort, the mean age (SD) at the index date was 85.74 (10.68) years. In terms of sex, 164 (80.39%) participants were male. Forty-two (20.58%) participants received <1 year of education, 81 (39.71%) had 1–6 years, and 81 (39.71%) had more than 6 years. In terms of marital status and smoking history, 123 (60.29) participants were married and 28 (13.73%) smoked. Thirty-five individuals (17.16%) had a history of diabetes mellitus, 85 (41.67%) had hypertension, 29 (14.22%) had cerebrovascular disease, 48 (23.53%) had heart disease, and five (2.45%) had hyperlipidemia. The crude prevalence rate of CI in the veterans group was 17.6% (18/102), whereas it was 19.6% (20/102) among the matched non-veterans.

**Table 1:** Baseline characteristics of old people with or without military employment before and after matching.

Characteristic	Before matching				After matching			
	*N* (%)	*N* (%)	SMD	*P*	*N* (%)	*N* (%)	SMD	*P*
	Veterans (*n* = 105)	Non-veterans (*n* = 11742)			Veterans (*n* = 102)	Non-veterans (*n* = 102)		
Age, Mean (SD), y	85.84 (10.64)	85.52 (11.91)	0.030	0.760	85.87 (10.55)	85.60 (10.85)	0.026	0.855
Female	19 (18.10)	6736 (57.37)	−1.020	<.001	19 (18.63)	21 (20.59)	−0.050	0.724
Education level, years				<.001				0.773
<1	22 (20.95)	5832 (49.67)	−0.706		22 (21.57)	20 (19.61)	0.048	
1–6	43 (40.95)	3717 (31.66)	0.189		42 (41.18)	39 (38.24)	0.060	
>6	40 (38.10)	2193 (18.68)	0.400		38 (37.25)	43 (42.16)	−0.101	
Marital status				0.001				0.595
Married	59 (56.19)	4584 (39.04)	0.346		58 (56.86)	65 (63.73)	−0.139	
Separated	4 (3.81)	200 (1.70)	0.110		3 (2.94)	3 (2.94)	0.000	
Divorced	0 (0.00)	33 (0.28)	−0.053		–	–	–	
Widowed	41 (39.05)	6827 (58.14)	−0.391		40 (39.22)	34 (33.33)	0.120	
Never married	1 (0.95)	98 (0.83)	0.012		1 (0.98)	0 (0.00)	0.100	
Smoking	16 (15.24)	1483 (12.63)	0.073	0.008	16 (15.69)	12 (11.76)	0.108	0.606
Diabetes	1119 (9.53)	21 (20.00)	0.262	<.001	18 (17.65)	17 (16.67)	0.026	0.582
Hypertension	42 (40.00)	4749 (40.44)	−0.009	0.961	39 (38.24)	46 (45.10)	−0.141	0.661
Cerebrovascular disease	15 (14.29)	1287 (10.96)	0.095	0.493	14 (13.73)	15 (14.71)	−0.028	0.571
Heart disease	22 (20.95)	1970 (16.78)	0.103	0.167	20 (19.61)	28 (27.45)	−0.198	0.218
Hyperlipidemia	3 (2.86)	629 (5.36)	−0.150	0.284	2 (1.96)	3 (2.94)	0.058	0.604

SMD, standardized mean difference.

Table 2 presents the results of the regression analyses that were conducted to evaluate the association between military employment and cognitive function. The following four models were generated: Model 1 included only the military employment status; Model 2 incorporated the demographic covariates; Model 3 further controlled for the lifestyle-related covariates; and Model 4 was further adjusted for chronic diseases. Military employment was not associated with the risk of cognitive impairment in any of the four models, all of which revealed that cognitive function declined with respect to age and the presence of hypertension.

**Table 2:** Associations between military employment and cognitive function for Chinese elderly (OR [95% CI])^a^.

	Model 1 (*n* = 204)	Model 2 (*n* = 204)	Model 3 (*n* = 184)	Model 4 (*n* = 174)
Military employment	0.88 (0.43∼1.78)	0.77 (0.34∼1.78)	0.79 (0.34∼1.83)	0.61 (0.24∼1.55)
Age		1.14*** (1.08∼1.21)	1.15*** (1.08∼1.22)	1.15*** (1.08∼1.23)
Female		0.96 (0.32∼2.84)	0.93 (0.31∼2.83)	1.15 (0.32∼4.16)
Education level		0.42 (0.14∼1.27)	0.38 (0.12∼1.18)	0.33 (0.09∼1.19)
Married		0.95 (0.07∼13.80)	0.88 (0.05∼14.03)	0.64 (0.03∼12.91)
Smoking			2.06 (0.48∼8.79)	2.22 (0.46∼10.66)
Diabetes				1.24 (0.27∼5.65)
Hypertension				0.26* (0.07∼0.99)
Cerebrovascular disease				5.07* (1.01∼25.64)
Heart disease				1.57 (0.36∼6.80)
Hyperlipidemia				5.34 (0.24∼116.69)

a cognitive impairment uses cut-offs: ≤16 points for those without schooling, ≤19 points for those with 1 to 6 years of education, and ≤23 points for those with > 6 years education.

* *p* < 0.05.

*** *p* < 0.001.

To verify the robustness of the results, sensitivity analyses were conducted in which cognitive impairment was defined according to the higher education-specific cut-off scores of the CMMSE (≤ 17 points for those without schooling; ≤20 points for those with 1–6 years of education; and ≤ 24 points for those with > 6 years of education). The analysis was repeated using Model 4 (Table 3), and the results were consistent with the initial findings.

**Table 3:** Associations between military employment and cognitive function for Chinese elderly (sensitivity analyses, OR [95% CI], *n* = 174)^a^.

Variables	Model 4
Military employment	0.64 (0.26∼1.59)
Age	1.15*** (1.07∼1.22)
Female	1.47 (0.42∼5.13)
Education level	0.28 (0.08∼0.99)
Married	0.47 (0.02∼10.19)
Smoking	2.57 (0.53∼12.48)
Diabetes	1.04 (0.23∼4.67)
Hypertension	0.26* (0.07∼0.96)
Cerebrovascular disease	6.02* (1.26∼28.80)
Heart disease	1.57 (0.38∼6.50)
Hyperlipidemia	3.35 (0.16∼71.03)

a cognitive impairment uses cut-offs: ≤17 points for those without schooling, ≤20 points for those with 1 to 6 years of education, and ≤24 points for those with > 6 years education.

* *p* < 0.05.

*** *p* < 0.001.

## Discussion

Military service represents a distinctive and formative life experience that exerts lasting influences on subsequent life trajectories. While existing research has extensively documented the physical and mental health outcomes associated with military employment (24–26), the specific impact on cognitive health remains an understudied area in the literature. This gap is particularly noteworthy given the unique occupational exposures and lifestyle patterns characteristic of military careers that may potentially influence long-term cognitive functioning. Cognitive impairment is common among military veterans, with an estimated prevalence of 3.3%–10% based on studies involving different objective and subjective (self-reported) cognitive assessments (27–29). The domains of cognitive function include sensation and perception, motor skills and construction, attention and concentration, memory, executive function, processing speed, and language/verbal skills (30). Cognitive impairment is characterized by a decline in one or more cognitive domains, representing a significant public health challenge with far-reaching consequences. This condition not only adversely impacts individual mortality and quality of life but also imposes substantial burdens on healthcare system, family and society (31). While numerous epidemiological studies have examined cognitive impairment patterns in general elderly populations, research specifically investigating potential differences in cognitive function between veterans and non-veterans remains limited. This represents a critical knowledge gap, given that military service constitutes a unique life experience that may influence neurocognitive aging through various biological, psychological, and social pathways.

As the rigorous sampling design and standard methods used in this cross-sectional study, we have provided evidence of the association between military employment and the risk of cognitive impairment in Chinese elderly adults. In this study, military employment was not significantly associated with the risk of cognitive impairment in any of the four models, including those in which adjustments were made for demographic, lifestyle, and chronic disease factors, and was consistent in sensitivity analyses. Previous findings regarding the association between military employment and cognitive decline or dementia have been inconsistent. For example, in a cohort study, veterans were found to exhibit a significantly lesser decrease in cognitive decline at follow-up assessments compared to non-veterans (32, 33). However, another study reported that military employment was not associated with an increased risk of either cognitive decline or dementia (34). These discrepant findings may reflect methodological variations in cognitive assessment approaches. The cognitive screening using CMMSE lacks the diagnostic precision of comprehensive neuropsychological evaluations required for formal cognitive impairment diagnosis.

Furthermore, the attenuation of the Healthy Soldier Effect (HSE) over time could potentially explain the convergence of cognitive outcomes between veteran and non-veteran populations in later life. Though the health of prospective military personnel is thoroughly screened prior to entering the military, some researchers have reported that the HSE may become less apparent and may even reverse course as veterans age, as evidenced by an elevated rate of mortality compared to expectations in older adults (15, 35, 36).

This pattern extends to cognitive trajectories, as demonstrated by the Health and Retirement Study (37). In that study veterans tended to experience better cognitive functioning relative to that of non-veterans around the age of retirement; however, they experienced an accelerated decline over time, resulting in no intergroup differences among those in the oldest age bracket. Several mediating factors may underlie this phenomenon, including educational attainment, marital status, and the cumulative burden of chronic diseases (34). These variables warrant particular attention in veteran populations, as they may significantly influence the long-term cognitive consequences of military service. Future research should prioritize longitudinal investigations to better elucidate these complex relationships and their implications for cognitive aging in veteran populations. Military-specific exposures represent another significant factor influencing veterans’ cognitive function (38). Extensive research indicates that combat exposure leads to substantially elevated rates of PTSD and depression in service members (39, 40). Both PTSD and depression have been consistently linked to an increased risk of cognitive decline and dementia in longitudinal studies, with these conditions often co-occurring, potentially compounding their detrimental effects on cognitive health. These findings highlight the complexity of studying military service as a life course determinant of cognitive health. Future research with more detailed characterization of military service experiences and longitudinal cognitive assessments may help clarify these relationships.

Although the analyses did not yield any evidence that military employment may have affected the risk of cognitive impairment in this sample, such dysfunctions may continue to be an important health condition that must be adequately addressed among the aging veterans represented in this study. A multicity study in China reported mild cognitive impairment in 2,027 veterans out of 5,496 participants (41), indicating that the prevalence in that particular sample was higher than that reported (1.3%) among U.S. veterans (42). In a randomly sampled survey of Chinese veterans, the prevalence of cognitive impairment was as high as 17.21% (43). Another nationwide survey of retired veteran cadres of the Chinese army reported that 45.8% of patients with Parkinson's disease (PD) had mild cognitive impairment and 31.3% had Parkinson's disease dementia (44). Chinese veterans with Parkinson's disease showed a greater rate of cognitive impairment. According to the 2024 White Paper on Veterans’ Services and Guarantees, China's veteran population currently stands at 57.8 million, with a mean age of 72 years. Given the aging demographic profile of this large population, the burden of cognitive impairment among Chinese veterans may increase substantially in the coming decades. The Chinese government has demonstrated a strong commitment to addressing dementia among the elderly population. In 2024, aligning with the World Health Organization's global initiatives, China launched the comprehensive “National Action Plan for Addressing Dementia in the Elderly (2024–2030)”. This strategic plan establishes a systematic framework for dementia prevention and treatment, marking significant progress in elderly healthcare (45). However, current policies continue to show insufficient focus on addressing cognitive impairment specifically among veteran populations, suggesting that further attention may be warranted if future nationally representative studies confirm an elevated burden in this group (41). To systematically address cognitive impairment, it is imperative to establish a comprehensive nationwide monitoring system. This system would serve three critical functions; (1) tracking the epidemiological patterns of cognitive impairment, (2) identifying key risk factors and determinants, and (3) informing targeted prevention strategies (46). For military veterans specifically, there is a pressing need to implement standardized protocols for early cognitive impairment screening, diagnosis, and intervention. Such measures would enable timely detection and treatment, significantly improving the potential for reversing cognitive dysfunction in its early stages (47). These initiatives would substantially strengthen the foundation for both primary prevention (reducing incidence) and secondary prevention (early detection and intervention).

This study had some limitations. First, the design was cross-sectional in nature, making it impossible to assess potential causal relationships between military employment and cognitive functioning. Second, it should be noted that the screening of cognitive impairment based on CMMSE scores is not equivalent to a formal diagnosis of cognitive impairment. Third, the results were restricted to Chinese veterans and may not be representative of other patient populations. Fourth, data were collected from the CLHLS, which did not always contain detailed information about military service. Fifth, the study relied on self-reported measures for chronic diseases including hypertension, hyperlipidemia, diabetes, heart disease, and cerebrovascular disease. This methodological limitation raises concerns about potential underdiagnosis of these conditions, which may consequently introduce unmeasured confounding variables into the analysis. Sixth, the matched analytic sample comprised only 102 pairs, which limited the statistical power to detect modest associations. *post-hoc* calculation indicated that, given the observed outcome rates of 17.6% among veterans and 19.6% among matched non-veterans, the minimum detectable odds ratio at 80% power and a two-sided alpha of 0.05 was approximately 3.0. Consequently, we cannot rule out the possibility of a true moderate association that was not captured owing to insufficient sample size. In the fully adjusted model, hypertension unexpectedly exhibited a protective association (OR = 0.26), which likely reflects multicollinearity and over adjustment rather than a true biological effect. Future investigations with larger matched or unmatched cohorts are warranted to clarify this relationship. Moreover, the CLHLS does not capture detailed information on military service experiences (e.g., duration of service, service branch, rank, combat exposure, deployment history, and age at service entry). Veteran status in this study was ascertained through self-report; therefore, it may be susceptible to non-differential misclassification, which could bias the effect estimates toward the null. Accordingly, the absence of a significant association should be interpreted with caution, as it may reflect either a true null effect or an attenuated estimate resulting from exposure misclassification. Finally, this study did not account for every factor that could potentially influence cognitive function, such as genetic predisposition or symptoms of mental health disorders.

## Conclusions

The results of this study indicated that military employment was not significantly associated with a greater risk of cognitive impairment later in life. However, elevated exposure to general and military-related risk factors may partially contribute to the high prevalence of cognitive impairment observed in Chinese veterans, which remains an important health condition that must be addressed in this patient population. Future studies are needed to elucidate the means by which various risk factors associated with military employment can affect the cognition of veterans later in life.

## Data Availability

The datasets used in this study are available from the CLHLS upon reasonable request, https://doi.org/10.18170/DVN/ WBO7LK.
