# Supplementary material for: Is military employment associated with the risk of cognitive impairment in older adult? Evidence from China
Source: Front Epidemiol. 2026 Jul 23;6:1873441. doi: 10.3389/fepid.2026.1873441 (PMC13442525; doi:10.3389/fepid.2026.1873441)
Supplement: Supplementary file 1 [file Table1.docx]

**Supplement 1:**  Multiple regression analysis results of factors associated with cognitive function for Chinese elderly (n=174)

| Variables | β | S.E | t | P | β (95%CI) |
| --- | --- | --- | --- | --- | --- |
|  |  |  |  |  |  |
| Intercept | 48.86 | 5.36 | 9.12 | <.001*** | 48.86 (38.37 ~ 59.36) |
| Military employment | 0.69 | 1.09 | 0.63 | 0.527 | 0.69 (-1.45 ~ 2.83) |
| Age | -0.28 | 0.06 | -4.74 | <.001*** | -0.28 (-0.40 ~ -0.17) |
| Female | -1.92 | 1.68 | -1.14 | 0.256 | -1.92 (-5.22 ~ 1.38) |
| Education level |  |  |  |  |  |
| 1-6 | 2.49 | 1.62 | 1.53 | 0.127 | 2.49 (-0.69 ~ 5.67) |
| >6 | 0.44 | 1.69 | 0.26 | 0.794 | 0.44 (-2.87 ~ 3.76) |
| Marital status |  |  |  |  |  |
| separated | 3.38 | 4.23 | 0.80 | 0.426 | 3.38 (-4.92 ~ 11.68) |
| widowed | -3.04 | 1.40 | -2.18 | 0.031* | -3.04 (-5.77 ~ -0.30) |
| never married | -26.40 | 7.27 | -3.63 | <.001*** | -26.40 (-40.64 ~ -12.15) |
| Smoking | -0.79 | 1.64 | -0.48 | 0.631 | -0.79 (-4.00 ~ 2.42) |
| Diabetes | 0.34 | 1.58 | 0.21 | 0.832 | 0.34 (-2.77 ~ 3.44) |
| Hypertension | 2.10 | 1.18 | 1.77 | 0.078 | 2.10 (-0.22 ~ 4.42) |
| Cerebrovascular disease | -2.96 | 1.70 | -1.74 | 0.084 | -2.96 (-6.30 ~ 0.38) |
| Heart disease | -0.34 | 1.39 | -0.25 | 0.805 | -0.34 (-3.06 ~ 2.38) |
| Hyperlipidaemia | -4.00 | 3.80 | -1.05 | 0.294 | -4.00 (-11.44 ~ 3.45) |
| * p < 0.05, ** p < 0.01, *** p < 0.001  CI: Confidence Interval | | | | | |

**Supplement 2:**  Distributions of propensity scores in the veteran and non-veteran groups before and after matching
